# Emergence of Monkeypox as the Most Important Orthopoxvirus Infection in Humans

**DOI:** 10.3389/fpubh.2018.00241

**Published:** 2018-09-04

**Authors:** Nikola Sklenovská, Marc Van Ranst

**Affiliations:** Laboratory of Clinical Virology, Department of Microbiology & Immunology, Rega Institute for Medical Research, KU Leuven, Leuven, Belgium

**Keywords:** monkeypox, *orthopoxvirus*, emerging infectious diseases, zoonosis, disease outbreaks, one health

## Abstract

Monkeypox is an emerging zoonotic disease recognized as the most important orthopoxvirus infection in humans in the smallpox post-eradication era. The clinical presentation of monkeypox is similar to the one of smallpox. The case fatality rate of monkeypox (10%) lies between the case fatality rate of variola major (30%) and variola minor (1%). The disease is endemic in the Democratic Republic of the Congo, but other countries of Central and West Africa either reported cases of monkeypox in humans or circulation in wildlife. The disease was also imported once into the USA. The disease has always been considered rare and self-limiting, however recent sporadic reports suggest otherwise. Unfortunately, the collected data is limited, dispersed and often incomplete. Therefore, the objective of this review is to trace all reported human monkeypox outbreaks and relevant epidemiological information. The frequency and geographical spread of human monkeypox cases have increased in recent years, and there are huge gaps in our understanding of the disease's emergence, epidemiology, and ecology. The monkeypox virus is considered a high threat pathogen causing a disease of public health importance. Therefore, there is an urgent need to focus on building surveillance capacities which will provide valuable information for designing appropriate prevention, preparedness and response activities.

## Introduction

Monkeypox (MPX) is an emerging zoonotic disease caused by *monkeypox virus* (MPXV), a member of the *Orthopoxvirus* genus in the *Poxviridae* family. MPXV is one of the four *Orthopoxvirus* species pathogenic for humans, together with *variola virus*, the causative agent of smallpox, now eradicated in nature; *cowpox virus*, and *vaccinia virus* (1). Monkeypox can infect a taxonomically wide range of mammalian species but the natural host is unknown. The virus has only been isolated twice from a wild animal, a rope squirrel in the Democratic Republic of Congo (DRC) (2) and a sooty mangabey in Ivory Coast (3). Transmission is believed to occur via saliva/respiratory excretions or contact with lesion exudate or crust material. Viral shedding via feces may represent another exposure source (4, 5). The clinical picture of monkeypox closely resembles the one of smallpox but the major difference distinguishing MPX from smallpox is the lymph node enlargement that occurs early, often at the onset of fever. A rash usually appears 1–3 days after the onset of fever and lymphadenopathy, with lesions appearing simultaneously, and evolving at a similar rate. Their distribution is mainly peripheral but can cover the whole body during a severe illness. The infection can last up to 4 weeks until the lesion desquamate (6). Patients can suffer from a range of complications including secondary bacterial infections, respiratory distress, bronchopneumonia, gastrointestinal involvement, dehydration, sepsis, encephalitis, and corneal infection with ensuing loss of vision. No specific treatment for a monkeypox virus infection currently exists, and patients are managed with supportive care and symptomatic treatment (7).

In an African setting, MPX can be misdiagnosed with other rash illnesses. The most common misdiagnosis [up to 50% of suspected MPX cases in the DRC (8, 9)] is chickenpox, also known as varicella, caused by *varicella zoster virus* (VZV). Coinfections with both MPXV and VZV were reported only a few times (8, 10, 11), however it was recently suggested that it is a relatively common phenomenon (12). The role of the VZV in MPXV epidemiology is not clear. Besides chickenpox, MPX can be misdiagnosed as cutaneous anthrax, fungal infection in HIV patients, *Staphylococcus* sp. related rash (13) or other diseases which cause rash.

Two genetic clades of *Monkeypox virus* have been characterized including the West African and the Central African clade. These two clades are geographically separated and have defined epidemiological and clinical differences. The West African clade demonstrates a case fatality rate (CFR) <1%, and no human-to-human transmission was ever documented. In comparison, the Congo Basin clade (also known as the Central African clade) show a CFR up to 11% (14), and documented human-to-human transmission up to 6 sequential events was observed. The isolates from the West African clade originated from outbreaks in Nigeria, Liberia, Ivory Coast, Sierra Leone, and USA (imported from Ghana) while the isolates belonging to the Central African clade came from Gabon, Cameroon, the Republic of Congo (ROC), Central African Republic (CAR), Sudan, and the DRC (14–17). According to available data, the Congo Basin clade is more common than the West African clade given it is endemic in the DRC where more than 2,000 suspected cases are reported every year (18). However, MPX is not part of mandatory reporting in other countries than the DRC, which might introduce a bias (19).

MPX has always been considered a rare sporadic disease with a limited capacity to spread between humans (20). Nonetheless, it is a life-threatening disease in the DRC and other countries of western and central Africa (8) and possibly worldwide. The threat would increase if there would be a virulence increase [both naturally (1, 21) or through genetic engineering (22)], a virus spill into more widely distributed taxa (23) or an introduction in other continents (24). Consequently, MPXV belongs to the “biosafety level 3” category, the “high threat” biodefence category in the EU (25) and is on the list of select agents in the USA (26).

The scientific community is hesitant to the importance of MPX which can be demonstrated by a limited number of research articles in the biomedical literature. The biggest number of articles was published in a year when MPX was reported outside of the African continent for the first time in history. In the past decades, we have seen a similar reluctance to research and control of other pathogens with epidemic potential (Zika and Ebola as recent examples) which were geographically limited to the Southern Hemisphere.

Monkeypox outbreaks are rarely reported, badly managed and little described leading to an incomplete picture of the disease's importance. MPX is the next most pathogenic poxvirus disease after smallpox but never received appropriate attention to prevent it to become an epidemic. The next sections attempt to summarize all publicly available literature on human monkeypox cases, both official and unofficial, since the first case in 1970 until 2018.

## Outbreaks of monkeypox in humans

### Official sources

**Figure 1:**
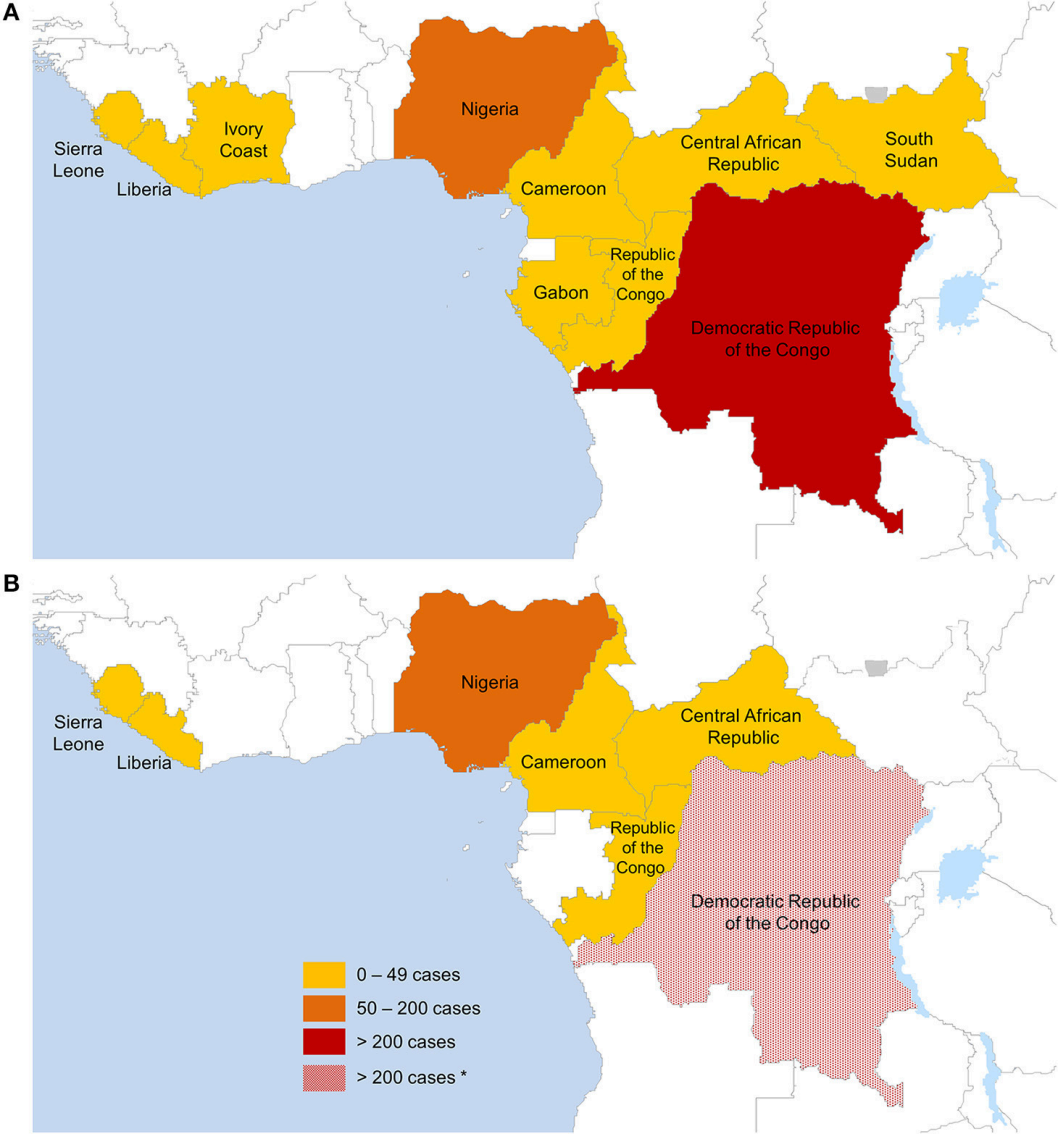
Countries reporting confirmed monkeypox cases in humans **(A)** between August 1970 and May 2018 (574 months), **(B)** between January 2017 and May 2018 (17 months). Only data from official sources were included. The lack of data granularity for some outbreaks does not allow displaying exact geographical outbreak locations. *Estimated cases based on available data on monkeypox confirmed cases from the DRC since 2005.

The outbreak overview below covers all cases published via official channels (research articles and reviews, books, WHO reports). All reported outbreaks discussed below are summarized in Table 1 and the distribution of confirmed cases per country is shown in Figure 1.

**Table 1:** Cases of human monkeypox reported in the World from 1970 till 2018.

	**1970-1990**	**1991-1999**	**2000-2009**	**2010-2018**
**Democratic Republic of Congo (former Zaire)**	386 (confirmed) (27) + 2–5 (28, 29)	511 (28, 30)	Not fully enumerable (8, 11, 24)	Not fully enumerable (18, 31–34)
**Central African Republic**	6 (confirmed) (35)	N/A^*^	4 (19)	At least 68 (at least 29 confirmed) (19, 34, 36–38)
**Cameroon**	2 (confirmed) (27, 39)	4 (1 confirmed) (28, 29, 40, 41)	N/A^*^	16 (1 confirmed) (34)
**Nigeria**	10 (3 confirmed) (42–44)	N/A^*^	N/A^*^	244 (101 confirmed) (34)
**Ivory Coast**	2 (confirmed) (43, 45)	N/A^*^	N/A^*^	N/A^*^
**Liberia**	4 (confirmed) (42, 46)	N/A^*^	N/A^*^	2 (confirmed) (19)
**Sierra Leone**	1 (confirmed) (42, 46)	N/A^*^	N/A^*^	At least 2 (2 confirmed) (19, 40, 47)
**Gabon**	1–10 (one confirmed) (28, 29, 40, 48)	N/A^*^	N/A^*^	N/A^*^
**USA**	N/A^*^	N/A^*^	47 (37 confirmed, 10 probable) (49, 50)	N/A^*^
**Republic of Congo**	N/A^*^	N/A^*^	12 (3 confirmed, 8 probable) (51, 52)	98 (9 confirmed) (53, 54)
**South Sudan**	N/A^*^	N/A^*^	49 (10 confirmed, 9 probable) (55)	N/A^*^

*Confirmation status mentioned between brackets if known*.

* No information available

#### 1970–1990

The first human MPX case was reported in August 1970 in Bokenda, a remote village in the Equatorial province of the DRC (called Zaire at the time). A 9 month old child was admitted to Basankusu Hospital with suspected smallpox (56) and a sample, sent to the WHO Smallpox Reference Centre (Moscow), revealed MPXV by virus isolation (57). The patient's family said that monkeys were eaten occasionally as a delicacy but they were not able to recall if monkeys had been eaten during the last month or if the child came into contact with a monkey lately. The investigation showed that the child was the only one in the family who had not been vaccinated against smallpox (56).

The first 20 human cases were described by the WHO (42, 58, 59) in 1972 and 1976, and 15 newly reported cases were included in the update of 1978 (60). Later, WHO reported 54 cases between 1970–1979 (20, 30, 61), out of which 47 were described by Breman et al. (43). JeŽek et al. reported 59 cases between 1970 and 1980 (27, 62). This number of 59 cases was also reported in one of the later reviews, but no reference was mentioned (28).

The Global Commission for the Certification of Smallpox Eradication designated MPX as the most important orthopoxvirus infection in men in the post-smallpox eradication era (from 1980). They recommended in their final report in 1980 that a surveillance program on MPXV and its epidemiology and ecology should be continued (63). After 1980, the number of reported cases among reports differ. Arita et al. reported 98 cases (61), a WHO meeting counted 100 cases (20) and JeŽek et al. reported 132 cases between 1981–1983 (62). Between 1980 and 1986, a total number of 350 (30) and 346 (62) cases were reported. The WHO active surveillance program between 1981 and 1986 identified 338 confirmed monkeypox cases and 33 deaths (CFR 9.8%). The increase in MPX cases between 1981 and 1986 was probably due to strengthened surveillance since there was no substantial increase in the number of cases coming from the areas not under the WHO surveillance (27).

Although the number of yearly reported cases in different countries varies among sources, the total number of confirmed MPX cases reported per country from 1970 till the end of 1986 is the same, resulting in 404 cases (30, 64). Some sources report 404 cases during the WHO surveillance period (1981–1986) alone, but without (6, 28) or with an incorrect reference (24).

Human-to-human transmission occurred occasionally from primary human cases but very rarely from secondary cases (61). There was no increase in the secondary attack rate for household contacts from the period 1970–1980 (12%) to the period 1981–1986 (9.28%) thus there was no evidence for a change in transmissibility of the virus (4). The longest reported chain of inter-human transmission consisted of four serial cases, which indicated little potential of the virus for epidemic spread (27, 65). Primary infections from animals were responsible for 72% while inter-human transmission accounted for 28% (29, 66). Children below 10 years of age accounted for the majority (86%) of cases (6, 27). Males (58%) predominated females (42%) among the primary cases (especially in the age group 5–14 years), while female patients were more common among the secondary patients. It showed that the unvaccinated young males were at greater risk for infection, probably because they trapped and played with small rodents and their carcasses. Females were at risk because of child-to-mother infections (27). Local population immunity from the smallpox vaccination was estimated at ≥60% during the 1970s and the early 1980s (29). Almost all primary cases occurred in unvaccinated individuals (average annual primary attack rate of 1.7 per 10,000, compared to a rate of 0.04 per 10,000 among those vaccinated) (27). Sera of 774 unvaccinated contacts of reported cases were taken during the WHO surveillance (1981–1986) and 136 (18%) of them had been infected with MPXV (27).

The number of reported cases dramatically decreased after the intensified surveillance was discontinued. Outbreaks were reported from **Gabon** in **1987 [**44] and the **DRC** (called Zaire at the time) between 1987 and 1992. However, the numbers of reported cases differ between sources and the year of the outbreak in the DRC is not clear (28, 29, 40, 48).

#### 1990-1999

Four cases of suspected monkeypox (1 confirmed case) were reported from **Cameroon in 1990 [**38, 45, 46], making them the third ever described monkeypox outbreak in Cameroon.

No cases were reported between 1993 and 1995 (28). A prolonged outbreak of human MPX in the **DRC** occurred during **1996** and **1997**. The first case was reported in mid-February 1996 in the village Akungula, but the outbreak became apparent only at the end of July when more people got infected. 71 suspected cases (out of which 11 were confirmed by a lab and 6 were fatal) were reported in 13 villages (Katako-Kombe health zone, Sankuru Sub-region, Kasai Oriental Region) until 30 August 1996. Investigation showed evidence of orthopoxviruses in all sera available (67, 68). During two following WHO investigations, large numbers of new suspected cases of MPX were reported (69).

In total, 511 suspected cases occurred in 54 villages in the Katako-Kombe health zone and 24 in the Lodja health zone between February 1996 and October 1997 (28, 69). The disease was relatively mild and the fatality rate was about 1–5% while the percentage of secondary cases was much higher than previously reported (about 78%). This suggested that a substantial proportion of the reported cases were VZV cases (70) which was later laboratory confirmed (10, 71). Some patients might have been coinfected with both MPXV and VZV since five MPXV-positive patients within one household showed serologic evidence of a recent VZV infection (and two of those had detectable VZV DNA in the skin lesion samples) (10). Moreover, a comparison of the isolated MPXV hemagglutinin sequence with the one from the 1970s suggested that the nucleotide sequence has not changed over the years (10). This outbreak represents the largest ever reported cluster of suspected cases spread over a large area of the Katako-Kombe and Lodja zones. This might have been caused by decreasing antibody protection of the local population since the smallpox vaccination cessation. Additionally, a period of civil unrest and economic collapse made them penetrate deeper and more frequently into the rain forest in search of food, thus increasing the risk of contact with animals carrying MPXV (10, 28, 69).

#### 2000-2009

No new suspected cases were reported until 2001 when, from **February till August 2001**, 31 patients in 7 separate disease clusters (outbreaks) were suspected in the Equateur province of the **DRC**. Specimens from 14 patients were tested out of which 6 were positive for MPXV, 5 for VZV, 1 positive for both viruses and 2 for none. There were 5 deaths reported of which 4 were confirmed to be caused by MPX (8).

Four cases of human monkeypox were reported from **CAR**, Mbomou region in **2001** (19, 72). No further information is available.

A report from health care workers in the **DRC** indicated that human cases of MPX were occurring more frequently than the few published articles would suggest (70, 73). A total of 1265 suspected cases were reported to the DRC Ministry of Health between **1 January 1998 and 31 December 2002**. The majority of these cases occurred in regions covered by the equatorial forest. 215 specimens (21.7%) were collected and 88 of these (41%) were confirmed as MPXV. More detailed epidemiological information was available for 56 MPXV-positive cases. Males were affected proportionally more than females (30 and 26 cases, respectively) and the most affected age group was 10–24 years of age accounting for 44.6% (compared to 28.6% for 0–9 years and 26.8% for 25 years and above). In a sample of 49 infected, 75.5% did not have a smallpox vaccination scar (73).

A report published later indicated that 2,734 cases of suspected human MPX were reported from all 11 **DRC** provinces during **January 2001–December 2004** as part of the DRC Ministry of Health national disease surveillance program. 380 cases were reported in 2001, 545 in 2002, 783 in 2003, and 1026 in 2004. The civil war impeded surveillance activities, therefore only 171 clinical specimens were obtained from 136 patients (representing only 4.9% of all reported cases). Out of these patients, 37.5% were MPXV positive, 44,8% VZV positive and 0.7% had a coinfection of both. Data on sex and age were available for 134 patients. The male-to-female ratio was approximately equal (66 males and 68 females). Ages ranged from 2 months to 54 years and the average was 15.4 years (median 11 years). The average age of patients with confirmed MPX was 10 years (median 7 years), whereas the mean age of patients with VZV infection was 20.6 (median 17.0, *p* < 0.001). That most MPX cases (94%) occurred in patients <25 years of age suggests that cross-protective immunity may still exist, however, this difference could also reflect different exposures in adults >25 years of age (11).

The first outbreak outside Africa happened in the **USA** in **2003** (49, 50, 74) following the import of infected animals from Ghana. The source of the outbreak was traced back to native prairie dogs (*Cynomys* spp.) housed with African rodents (*Funiscuirus* spp., *Heliosciurus* spp., *Cricetomys* spp., *Atherurus* spp., *Graphiurus* spp., and *Hybomys* spp.) that infected the prairie dogs at an Illinois pet distributor (74, 75). The laboratory investigation showed 3 virus-positive African species: *Cricetomys* spp., *Funisciuris* spp., and *Graphiuris* spp. (76). The outbreak counted 47 patients, with confirmed (37 cases) and probable (10 cases) MPXV infection (77) which occurred in Illinois, Indiana, Kansas, Missouri, and Wisconsin state (49). The manifestation of illness in this outbreak was relatively mild with some exceptions (50, 78, 79). There were no deaths, no person-to-person transmission could be proven (75), and it seemed that age and vaccination status had little effect on the clinical disease manifestation (nearly one third of infected adults received the smallpox vaccine before 1972) (80). Interestingly, it was the manner in which a person was exposed to MPXV (invasive or non-invasive) that had a greater effect (77). In contrast to MPX outbreaks in Africa that affected a disproportionate number of children, a majority of cases in the USA outbreak occurred in adults (80). Genetic analysis of the virus indicated that the strain belonged to the West African clade (50) which is probably the reason for a mild disease manifestation and no deaths.

The **ROC** reported a MPX outbreak in **April 2003** from the town Impfondo in the remote and heavily forested district of Likouala (51). This is the first reported outbreak of MPX in the country, however, the virus circulation was suggested earlier by a seroprevalence study in 1981 (81). In 2003, 12 cases (3 confirmed, 8 probable, and 1 suspected) were observed during this outbreak and all (except of the suspected case) were less than 18 years old (51, 52). Person-to-person transmission was prominent during the outbreak and the illness manifestations were relatively severe. One death occurred during this outbreak. The investigations support the hypothesis that at least seven virus transmission generations (six serial transmissions) occurred, which is the longest chain of transmission ever documented. This could be caused either by intense exposures or highly efficient transmission (51).

During **September-December 2005**, 10 confirmed, 9 probable, and 30 suspected MPX cases were reported from 5 villages (2 in Bentiu, 3 in Modin, 5 in Nuria, 5 in Rubkona, and 4 in Wang Kay) in Unity State, **Sudan** (area that is now part of South Sudan). However, the scope of this outbreak is believed to be underestimated. This was the first time MPX has been reported outside of its traditional ecology (tropical rainforest)—-in a dry savannah environment in Africa. Person-to-person transmission up to 5 generations were described and no deaths occurred during this outbreak (13, 55). It was postulated that the MPXV strain isolated during this outbreak has a novel genomic structural variation related to the Congo Basin. Furthermore, the presence of rodent hosts suggests that the virus could be endemic in the area (13). However, Nakazawa et al suggest that the 10.8–kb sequence duplicate represents a single mutation event and is not sufficient evidence to suggest an independent evolutionary trajectory given the Sudan isolate's overall similarity to the Congo Basin isolates. Their results support a hypothesis that the virus was imported, most likely from northern DRC (82).

The active MPX surveillance program in the **DRC** from **November 2005 till November 2007** identified 760 laboratory-confirmed cases of MPX in nine participating health zones. The highest incidence was recorded in zones with the greatest forest coverage (Kole, Lomela, and Tshudi Loto) and no detectable seasonality was observed. The average age of cases was 11.9 years, with the youngest 5 days and the oldest 70 years. Almost all (92.1%) of the cases were born after the mass smallpox vaccination campaigns (after 1980). There were significantly more male (62.1%) than female cases. When comparing the results of this surveillance study with the one in the 1980s, the average annual incidence in Kole showed a 20-fold increase (from 0.7 to 14.4 per 10,000) and the other zones indicate the same trend. These results are likely to be an underestimation because of the relatively limited resources which would allow the team to reach remote locations, the lack of diagnostic capacity and less funding and manpower than the surveillance in the 1980s (24).

#### 2010-2018

In **2010**, an outbreak in the **ROC** (Likouala region) accounted for 10 cases (2 confirmed, 8 suspected). This outbreak is thought to be associated with the movement of DRC refugees across the Ubangi River into the ROC after interethnic violence in north-western DRC. The cases were recognized thanks to a broad-based campaign to improve health standards in refugee settlement areas which included modules on monkeypox recognition and prevention ^68^ highlighting the benefits of active surveillance activities. Sequencing and phylogenetic analysis of the virus isolate (81137 nucleotide section, from E9L to A24R) closely resembled (~99.5%) the one recovered from the outbreak in the ROC in 2003 (51) and was less similar to the one from the DRC in 1979 and 1996 (10, 43).

In June of the same year (**2010**), two cases of MPX were confirmed in the **CAR**. The disease developed after hunting and eating a wild rodent (*bemba*). The isolated strain was identical to the one associated with an outbreak in 2001 on the border between the CAR and the DRC (36).

Data of the **DRC** Ministry of Health from **2010-2014** showed that the number of reported cases exceeded 2000 cases every year. At least one suspected case was reported from every region of the DRC while the Equateur and Kasai Orientale provinces have the biggest number of cases (1166 and 708, respectively). The reason why the Equateur province reports such a high number of suspected cases is probably because of the existence of a CDC project in the Tshuapa district (18, 31). 423 samples were collected from the suspected cases reported in 2014 from different provinces. The results showed that 62% (264 samples) were positive for orthopoxvirus, 15% (63 samples) were positive for VZV and 22% (92 samples) were negative for both viruses. This is a big increase compared to the years before 2005, and the disease occurred in provinces where it has not been reported before (18). Another source reports a total of 104 suspected cases with human MPXV infection and 10 deaths (CFR 9.6%) from the Bokungu Health Zone to the national surveillance system during **2013**. 60 (57.7%) patients had active lesions and their specimen were tested. 83.3% were MPXV-positive, 8.3% VZV-positive and 8.3% were negative for both viruses (32). Moreover, 6 cases of suspected MPX were reported from the forested areas of North (4 cases) and South Kivu (2 cases) provinces **from 2011 till August 2014**, areas from which reporting of MPX cases is really scarce. Two of the cases from North Kivu and one case from South Kivu were positive for MPXV, one of the cases from North Kivu was positive for VZV, and one case each from North and South Kivu had negative findings for both MPXV and VZV using a PCR diagnostic assay (33). To what extent the above three data sources overlap cannot be determined.

In **2014**, a MPX outbreak was reported in **Sierra Leone** (Bo City) after more than 40 years. Only one case was lab confirmed (40, 47) and the total number of infected people is not known.

From **December 2015 till February 2016**, at least 12 people (out of which 3 died) got infected with MPXV in Bangassou, Mbomou province, the **CAR**. The disease was confirmed by the laboratory of the Ministry of Health (37, 72, 83). Journal de Bangui (72) stated in the report announcing this outbreak that there was recently a mokeypox outbreak 2014 in Haute Kotto zone. There is no further information to support this so the validity cannot be confirmed.

**CAR** (Basse-Kotto and Haute-Kotto provinces) experienced at least 26 more suspected cases (of which at least 3 were confirmed) as of **August 2016** (38, 84).

Between **September 2014 and February 2016**, 587 suspected MPX cases were reported through a passive surveillance program of the **DRC**. The majority of reported cases were located in the Equateur province (363 cases) and the Tshuapa province (135 cases). The other provinces accounted each for 18 or less cases during this period. 55% of all suspected cases were male. 320 suspected cases were younger than 15 years, 256 were 15 or older. 592 clinical specimens were obtained from 339 patients which represents 57.8% of all reported suspected cases. 223 tested positive for MPXV, 40 for VZV and 78 for neither. There were two cases of coinfection with both, MPX and VZV (85).

Between **January and August 2017**, **ROC**, Likouala province reported monkeypox outbreak accounting for 88 cases out of which 7 were laboratory confirmed, including 6 deaths (CFR 6.8%). A total of 18 villages in 5 districts (Enyelle, Betou, Dongou, Impfondo, and Owando) have been affected (54). The outbreak was formally declared by the Congo state authority on 13th March. Children under 15 years of age were the most affected, accounting for 60% of the overall caseload. The gender distribution was proportionate, with 51% of the cases being female (86). This was the biggest outbreak of monkeypox reported in the ROC with high transmission potential probably due to prevalent underlying exposure risk factors in the communities. WHO considered the overall risk of this outbreak high at national level given the weak surveillance system coupled with the limited public health infrastructure. In addition, the risk of disease spreading to the neighboring countries was also considered high in view of the high population mobility and the presence of refugees from Central African Republic, Democratic Republic of Congo, and Chad (86). Monkeypox occurs in African countries which often have weak surveillance and public health infrastructure making the prevention and response difficult.

Two outbreaks were reported in **CAR** in 2017. The first outbreak was reported in Mbomou province in **February 2017**. Limited information is available for this event. 47 cases (5 confirmed) were reported by WHO in the bulletin of week 22 (87) however only 5 cases (2 confirmed) were reported in the bulletin of week 38 (88). No deaths were reported. The second outbreak was reported in Mbaki district in **April 2017** accounting for 3 cases, out of which 1 was laboratory confirmed. No deaths were reported. Further investigations supported by the Ministry of Health and WHO revealed that 24 of 26 (92.3%) of close contacts had antibodies (IgG) against monkeypox, and 4 against cowpox. This suggests a high level of circulation of the virus in the region, and may explain the low number of cases recorded during these outbreaks (89).

One isolated case of monkepox was confirmed in **Sierra Leone** (Pujehan District) in March **2017**. Thirteen close contacts were followed up and none of them have developed any febrile illness and/or skin lesions in the first 21 days since the last exposure (90).

From **September 2017 through April 2018**, 244 cases including 101 confirmed cases were geographically spread across 25 states and the Federal Capital Territory (FCT) of **Nigeria**. The confirmed cases were reported from 15 (out of 36) states (34). Six deaths were recorded (CFR 2.5). From September to December 2017, the majority of cases were male (75%) and aged 21–40 years old (median 30 years). (34, 91). The investigation established that the initial cluster of cases (two brothers, their uncle, and a neighbor) fell sick after killing and eating a captured monkey from the neighborhood, which young boys regularly played with (92). Phylogenetic analysis indicated that the closest relative of this outbreak isolates were two Nigerian strains isolated in 1971, belonging to the west African clade (44). This outbreak is the largest documented outbreak of human monkeypox in West Africa.

Sixteen cases of monkeypox, including 2 confirmed cases were reported in **Liberia** between **November and December 2017**, after more than 40 years from the last (and the first) reported occurrence. The cases were reported from Grand Cape Mount (4), Rivercress (11), and Maryland (1). Two deaths were recorded (CFR 12,5%) (93).

Between **week 1 and 24 of 2018**, there have been 2845 suspected cases of monkeypox in 14 provinces of the **DRC**, including 36 deaths (CFR 1.3%). Of the suspected cases, 34 have been confirmed samples (34). Sankuru Province has had an exceptionally high number of cases between week 1 and 14 of this year (106 cases) compared to the same time period last year (44 cases) (94).

Between **17 March and 24 April 2018**, 20 cases of monkeypox were reported in **CAR**, including 9 cases confirmed by the laboratory (6 cases from Ippy, 3 cases from Bangassou).No deaths were recorded (34).

A total of 16 monkeypox cases have been reported in **Cameroon** between **30 April and 30 May 2018**, including 1 confirmed case and no deaths. The cases were recorded in Njikwa Health district (7, including 1 confirmed), Akwaya Health District (6), Biyem-Assi health district (1), Bertoua Health District (1), and Fotokol Health District (1) (34).

### Unofficial sources

Unofficial sources like ProMED mail or other reporting systems of outbreaks are rapid and important sources of information. These facilitate public health professionals to respond and control disease outbreak. Nevertheless, the validity of the unofficial notification is not guaranteed and detailed epidemiological information is often missing. Data from unofficial sources is summarized in Table 2.

**Table 2:** Cases of human monkeypox summarized from ProMED reporting system.

**Start of outbreak**	**Number of cases**	**Where**	**Notes**	**ProMED archive number, reference**
February 2016	195 (8 deaths)	Bas-Uele province, DRC	Not confirmed	20160212.4014697 (95, 96)
September 2015	At least 20	Tshuapa province, DRC	Samples sent for testing, no further details	20150914.3643887 (97)
June 2014	12 (2 deaths)	Bomongo territory, DRC	Not confirmed	20140621.2557186 (98)
September 2012	1	Butembo, North Kivu province, DRC	Not confirmed	20120917.1297716
January 2011	At least 114 (5 death)	At least 3 health zones of the Equateur province, DRC	At least 3 cases were **confirmed** by a lab. A certain proportion likely to be misdiagnosed with varicella.	20110113.0148 20110221.0569 (99)
July 2008	39 (includes 19 children from 0 to 5 years, 3 deaths)	Bokungu, DRC	Not confirmed	20080714.2141 (100)
September 2007	62	Likouala department, ROC	**Confirmed**. Most of the affected were refugees from the DRC younger than 15 years. According to testimonies from the villager, there were at least 150 cases in the district.	20070925.3181 (101)
August–October 2006	Not known	Dekese, Kasai Occidental region, DRC	Not confirmed	20061023.3040 (102)
May 2005	56 (no death)	Mbuji Mayi, South-Central region, DRC	Not confirmed	20050509.1276
October 2001–October 2002	485 (25 deaths) reported in the first six reports and the seventh report indicated 32 extra infected people (12 deaths)	6 foci (two in the North Equateur and 4 in the Equateur region), DRC	2 cases were **confirmed** by a lab. The number of cases is probably over-reported due to concomitant varicella (chickenpox) activity. A number of samples were sent for testing, no further details	20020228.3654 20020314.3744 20020315.3749 20020322.3797 20020409.3918 20020410.3926 20021025.5638
February 2001	Not known	Equateur Province, DRC	MSF France was reported to be collecting samples for lab testing, no further details	20010315.0523
During the year 1999	Not known (315 deaths)	Mbuji-Mayi, DRC	Not confirmed Unlikely to be only monkeypox cases (if there are any)	20000428.0645 20000506.0691

## Discussion

Monkeypox has already occurred in 10 African countries and is known to be endemic in the DRC. It has once crossed the borders of the African continent when it was imported into the USA in 2003, before which it was assumed that the disease was geographically limited. The summary of recorded cases above demonstrates an increase in the incidence of monkeypox cases in recent years, together with a broader geographical occurrence. However, the data collected is often incomplete and unconfirmed which hampers realistic estimations of prevalence and incidence of monkeypox over time. Nevertheless, the active surveillance conducted by Rimoin et al. between 2005 and 2007 concluded that there was a 20-fold increase in monkeypox incidence compared to the historic data between 1981 and 1986 (WHO active surveillance) (24). Also other official reports (e.g., DRC's passive surveillance program 2010-2016 data, see above) and unofficial source data (Table 2) underline the emerging character of the disease. Four explanations, possibly occurring simultaneously, were raised for the monkeypox incidence increase (24):

1. *The cessation of the smallpox vaccination in 1980 and the consequent drop of immunity against orthopoxviruses*. Eradication of smallpox and waning immunity against after vaccine cessation created an immunologic niche for monkeypox. In 2010, only 24.5% of the DRC population had evidence of a smallpox vaccination scar. The influence of vaccination cessation is reflected in the age pattern of incidence (24, 103). After 1980, more individuals are susceptible to MPXV infection every year. Similar causality was shown for measles (104) and yellow fever (105) when a decreased vaccination coverage led to increased incidence.
2. *Higher or more frequent exposure to animal reservoir species*. Significant anthropogenic and demographic changes have occurred in the DRC since 1980, and may have increased the exposure of the local population to reservoir species of MPXV. Logging of the rain forest increases the opportunities of human exposure to displaced animals, thus for zoonotic transmission of MPXV. Additionally, recurrent war, civil unrest and poverty force the affected population to flee and eventually look for shelter deeper in the rain forest, and to rely more extensively on bush meat (monkeys, small rodents, etc) (24, 106). Similar conclusions were drawn from studies on Nipah virus (107) and rabies virus (108) where environmental and anthropogenic changes led to a higher chance of exposure to (infected) animals, leading to increased incidence.
3. *Increased human-to-human transmission rate*. Increased prevalence in humans, particularly immunocompromised hosts, may provide more opportunity for MPXV to acquire mutations that increase its fitness in human hosts, possibly leading to increased transmissibility, virulence, and pathogenic potential. Also probabilistic arguments suggest that a zoonotic pathogen with an R_0_ near to one (such as monkeypox) retains a greater potential to evolve to a state of higher transmissibility as transmission chains lengthen and as the number of primary introductions increases (16, 24, 109). This assumes that the population lacks the vaccine-derived immunity, as it is the case nowadays because the smallpox vaccination was ceased (24). It needs to be studied whether the rising monkeypox incidence is a result of increased human-to-human transmission because greater circulation among humans opens the possibility of geographic spread by travelers (24). To demonstrate, a novel H1N1 influenza virus [A(H1N1)pdm09] crossed the species barrier from swine into humans in 2009 and multiple mutations during human-to-human transmissions allowed incremental changes in viral fitness, which may have contributed to the apparent increase in disease severity (110).
4. *Advancement in diagnostic capacity and health education*. Development of molecular techniques (47, 111, 112) and point-of-care systems (113) in recent years made the diagnosis of monkeypox more accurate and rapid than before. Also, more attention is paid to health education of medical workers and the general public (114). Despite these improvements which could cause higher reporting and confirming of cases, modern molecular techniques are more expensive (115), while today's surveillance programs have less funding and manpower resources than the WHO active surveillance program between 1981 and 1986. That is why the few active surveillance programs conducted nowadays are likely to be conservative estimates of a true monkeypox incidence increase (24).

The majority of data we currently have come from passive surveillance which often miss a proportion of cases, leading to an underestimation of the burden of MPXV infections in humans (18, 24). Despite this, recent research papers/reports based on passive surveillance (even though limited in number) provide evidence of the frequent occurrence of human MPXV infections (18). The incidence of monkeypox clearly increased but no detailed data is available to evaluate whether other aspects, like human-to-human transmission rate, morbidity and mortality rates or patterns of transmission changed (116).

The emergence of monkeypox as a significant human pathogen is indisputably a realistic scenario. Firstly, poxviruses were shown to be capable to rapidly adapt against host defenses despite their low mutation rates (117). Secondly, multiple countries were projected to have a suitable environment for MPXV by ecological niche modeling (82) where MPXV might be circulating undetected in animal hosts. In these countries, evolutionary, ecological, or epidemiologic changes could tip the balance in favor of emergence and possibly sustained transmission (23). A good example is Ghana where MPXV circulates in animals (118) but the country never reported human cases of MPX. The importation of MPXV-infected rodents to the USA, however, did lead to a human outbreak of MPX (50).

It is clear that surveillance but also research on the ecology, epidemiology, natural history and pathogenesis of the infection has to be addressed in order to design and implement needed prevention and control measures. Additionally, specific improvements in laboratory diagnostics and infection control measures are needed to detect cases, treat patients, and prevent further spread of the virus.

Because monkeypox is a viral zoonosis, coordination of interventions between the human and animal (wildlife) health sectors is necessary, including routine sharing of information (19). Data sharing does not only imply the animal-health sector in the context of One Health, but also inter-organization sharing as well as information sharing with the wider scientific community. Timely report publishing as a form of information sharing is a moral obligation of all health professionals. Without this common source of knowledge, there is no way to verify data obtained through passive, and in lesser amounts, active surveillance programs.

In the long run, comprehensive strategies to strengthen the health systems need to be initiated. This will enhance their resilience, in addition to building a strong preparedness and response capacity, thus avoiding outbreaks of big magnitudes in the future (86).

## Conclusion

It is time that we start to take action for prevention and preparedness of epidemics, especially for pathogens we have recognized as significant human threats, like monkeypox. The available data in this review demonstrate how limited and fragmented the information about monkeypox epidemiology still is, which leads to a potential underestimation of the magnitude and severity of MPX outbreaks. This should be a wakeup call to the research community for more engagement, follow-up and research on this disease. Despite being discovered in 1958 and for the first time described in a human in 1970, there are no standard guidelines for clinical management, nor therapeutics or vaccines (23). It is also an urgent call to leading public health institutions to take their responsibility to share data with health professionals and to communicate transparently to the public as soon as it becomes possible.

Monkeypox is a significant health concern for people living in endemic regions such as DRC and other African countries where circulation of the virus is confirmed, but it is also a global health security concern as demonstrated during the USA outbreak in 2003. Appropriate and effective interventions and active surveillance activities are urgently needed to prevent increased transmission efficiency or virulence (23). MPX is the most important orthopoxvirus in humans, certainly in the endemic areas and perhaps globally. Monkeypox is not a rare disease anymore, monkeypox needs more attention.

## Author contributions

NS and MV contributed to the conception and design of this review. NS was responsible for data collection, validation and analysis, and drafting of the manuscript. NS and MV edited and revised the final manuscript.

### Conflict of interest statement

The authors declare that the research was conducted in the absence of any commercial or financial relationships that could be construed as a potential conflict of interest.
